# The association between the frequency of physical activity and social background within and across home, occupational, transportation and leisure domains

**DOI:** 10.3389/fspor.2025.1609485

**Published:** 2025-08-12

**Authors:** Louise Briand Thomsen, Eva Berthelsen Schmidt, Birgitte Westerskov Dalgas, Karsten Elmose-Østerlund

**Affiliations:** Centre for Sports, Health, and Civil Society, Research Unit for Active Living, Department of Sports Science and Clinical Biomechanics, University of Southern Denmark, Odense, Denmark

**Keywords:** sport participation, exercise, training, active transport, work, household, active living, socio-economic status

## Abstract

This study investigates how often adults engage in physical activity across four life domains. Home, occupational, transport, and leisure using survey data from 163,000 Danes aged 15 and older. Activities were grouped into eleven categories, including housework, active transport, various types of training, sports, and outdoor activities. Descriptive statistics and multiple linear regression were used to examine patterns of participation and their association with social background, guided by ecological systems theory. Results show cumulative participation across activity types and significant variation linked to social background. The findings highlight the importance of distinguishing both between domains and between activity types within each domain when analyzing physical activity patterns.

## 1 Introduction

Physical activity (PA) encompasses a wide range of activities from competitive sports to everyday tasks. According to the World Health Organization's definition (1), PA includes all bodily movements produced by skeletal muscles that result in energy expenditure. Ecological models categorise PA into four domains: home, occupational, transportation and leisure (2, 3). The home domain encompasses domestic chores, such as gardening or vacuuming. The occupational domain relates to work or school activities, including jobs that require physical effort or school-based physical education. The transportation domain covers movements between places, such as walking to shops or biking to work. Lastly, the leisure domain includes voluntary activities undertaken during free time for enjoyment, relaxation or health benefits, such as jogging, swimming or playing various sports. Each of these domains is shaped by distinct combinations of individual, social, environmental, and policy-related fact (4), highlighting the need for domain-specific approaches in understanding and promoting physical activity.

Previous research has highlighted that each domain plays an important role in contributing to overall PA levels (8). A substantial portion of individuals’ moderate-to-vigorous PA comes from occupational, transportation and home tasks (5, 6, 7, 9). Conversely, leisure activities tend to contribute the least to overall PA levels (8, 9).

The conceptualisation of PA through an ecological lens emphasises that behaviours do not occur in isolation but are embedded within broader social and environmental contexts. Drawing upon ecological systems theory (10) and its applications in public health (4), ecological models posit that PA is shaped by multiple, interacting levels of influence, ranging from individual attributes (e.g., biological, psychological factors) to interpersonal relationships (e.g., family and peers), organisational settings (e.g., schools, workplaces), community environments (e.g., neighbourhood safety, built environment), and broader policy contexts (e.g., urban planning, transport policy, labour regulations).

Applying this framework to PA domains highlights that each domain is characterised by a unique constellation of determinants. For instance, leisure-time PA is often associated with individual motivation, self-efficacy, and access to recreational spaces, while occupational PA is shaped by job demands, workplace norms, and organisational health policies. Transportation-related PA is influenced by urban design, infrastructure, and safety perceptions, whereas home-based PA is embedded within domestic roles, household structures, and cultural expectations regarding gender and caregiving responsibilities (8). Garcia et al. (8) emphasise how environmental contexts, cultural perceptions, and social support uniquely influence each PA domain. However, despite the importance of all four domains, a systematic review indicates that most research to date has focused primarily on leisure and transportation activities, leaving occupational and home-based PA significantly understudied (8). Understanding how PA patterns differ by domain and social background thus remains critical for addressing health inequalities and developing targeted public health strategies (8).

Existing research has significantly advanced our understanding of how social determinants influence physical activity (PA) across leisure, occupational, transportation, and household domains. Home PA remains constant across age groups, and leisure PA tends to decline with age (11). Occupational PA is more common among individuals with lower socioeconomic status (SES) because people with higher SES are more likely to have sedentary jobs. In low-income and middle-income countries, occupational, household and transportation domains account for the majority of the typical PA, whereas leisure activities contribute more to total physical activity in high-income countries (12). Similarly, Cusatis and Garbarski (13) demonstrated that socioeconomic status (SES) significantly shapes domain-specific engagement, with individuals in lower SES groups typically reporting less leisure-time PA but higher levels of occupational and household activity [Cusatis & Garbarski]. This pattern is consistent with Beenackers et al. (14), who found that individuals with higher SES tend to engage in more PA during leisure compared to individuals with lower SES. However, as Holtermann et al.'s (15) highlighed through the concept of the ‘physical activity paradox’, in which occupational physical activity —more common among lower SES groups—does not confer the same cardiovascular health benefits as leisure-time physical activity, underscoring important health inequalities linked to domain-specific PA (15). Cillekens et al. (16) emphasise that while the health benefits of leisure-time physical activity persist across all occupational activity levels, they are particularly pronounced for individuals with sedentary jobs. However, workers in physically demanding occupations may face barriers to incorporating leisure-time activities due to long hours, insufficient recovery, and the physical demands of their work, underscoring the need for context-sensitive health promotion strategies. Vinluan et al. (17) found no gender-specific differences in PA levels in transportation or occupation, but home PA levels are higher among women whereas men are more inclined toward leisure activities.

The interaction between PA domains is complex, with research indicating both cumulative and compensatory patterns. In some cases, PA in one domain correlates positively with PA in others, suggesting a cumulative effect that supports integrated domain-based interventions (18–20). In other instances, a compensatory effect emerges, with individuals offsetting sedentary behaviour in one domain—often occupational—with increased activity in another, such as household or transportation activities (21, 22).

Despite existing evidence highlighting the importance of each life domain in contributing to overall physical activity, current knowledge remains fragmented in three key ways. First, prior studies have predominantly focused on leisure and transportation activities, often neglecting occupational and home-based domains (8). This limits understanding of how these understudied domains contribute to overall activity patterns. Second, while research has established associations between socioeconomic status and domain-specific physical activity patterns (13, 14) few studies have comprehensively analysed participation across all four domains within a single, large population sample. This restricts insight into how behaviours cluster within and across domains, and whether certain groups rely more heavily on specific domains for their activity. Third, most existing studies categorise physical activity broadly within each domain without differentiating between specific activity types, such as indoor vs. outdoor home activities or different forms of leisure-time exercise. This overlooks nuanced behavioural patterns that may have distinct determinants, health implications, and intervention potentials.

Additionally, there is limited knowledge of how social background characteristics, such as socioeconomic status, gender, or age, shape participation in specific types of activities within each domain. This constrains the ability to design public health interventions that address inequalities in physical activity by targeting domains and activities most relevant to different population groups.

To address these gaps, this study uses data from a large-scale survey of more than 163,000 adult Danes aged 15 years and older to investigate the frequency of physical activity participation within and across all four domains. By subdividing the domains into eleven specific activity groups—including housework, active transport, different types of training, sports, and outdoor activities—this study enables detailed analysis of how participation varies not only between domains but also between activity types within domains, and how these patterns are associated with social background characteristics. This comprehensive approach provides novel insights into domain- and activity-specific participation patterns, advancing understanding needed to develop targeted, context-sensitive, and equitable public health strategies.

This article examines two research questions (RQ). RQ1: What characterises the frequency of participation in physical activity in activities within and across the four life domains (home, occupational, transportation and leisure)? And RQ2: What characterises the associations between characteristics of social background and the frequency of participation in physical activity within and across the four life domains?

## 2 Methods

### 2.1 Data collection

This article uses data from a survey study from 2020 on the PA habits among Danish adults aged 15 or older. The survey population was stratified based on the size of their municipality before sampling. To gather reliable statistical data for all 98 Danish municipalities, a higher number of individuals from the smallest municipalities was sampled whereas a lower number was sampled from the largest municipalities. Based on the principle of stratified random sampling, a sample of 404,452 adults was drawn by Statistics Denmark.

The survey was distributed online via the digital mail system (‘e-Boks’) used for secure communication between citizens and public authorities in Denmark. The response rate was 40 per cent. A total of 143,794 adults answered all questions related to PA in the four domains and the social background variables. In order to account for the stratified selection procedure and non-response bias, we weighted the data in relation to gender, age and municipality size. The stratified random sample selection along with the application of survey weights increase the generalisability of our results, and, thereby, the validity of inferences made from our study towards the population of adult Danes aged 15 or older.

The survey contained questions on what kind of PA the participants practiced and how often they were active in different activities within the four domains of PA. The survey was validated through expert review, cognitive interviews, and piloting prior to distribution. A supplementary file presents the questions included in the questionnaire [see Supplementary File S1].

### 2.2 Dependent variables

The dependent variables correspond to activity groups within the four domains of PA and reflect the frequency of the respondents’ participation in PA, measured by the number of days per week they were active.

In the home domain, participants were asked about their frequency of various activities. The home domains were recoded into two categories for this analysis: *home indoor*, comprising the variables (1) cooking, tidying up and dishwashing, (2) cleaning and (3) doing laundry; and *home outdoor*, comprising the variables (4) gardening and (5) other practical tasks.

In the occupation domain, participants were asked about the level of physical exertion involved in their occupation. The occupation domain was categorised into one variable, *occupational PA*, which comprised answers to questions about how often their tasks involved (1) lighter physical exertion, (2) moderate physical exertion or (3) hard physical exertion.

In the transportation domain, participants were asked about the frequency of their active transportation. This domain was categorised into two variables: *walking as transportation*, comprising the variables (1) walking to and from work or study and (2) walking to and from other destinations; and *biking as transportation*, comprising the variables (3) biking to and from work or study and 4) biking to and from other destinations. In this survey, ‘other destinations’ was exemplified with grocery stores, childcare institutions, visits to friends and family, etc.

In the leisure domain, participants were asked first which activity types they had performed in their leisure time during the past 12 months and subsequently how often they had performed them. The questionnaire included 15 activity groups, which were recoded into six activity groups that represented the leisure domain in our analysis: (1) walking/biking, (2) running/fitness/flexibiliy training, (3) ball games, (4) gymnastics/dance, (5) water/outdoor activities and (6) other sport/street activities. Table 1 presents an overview of the dependent variables (activity groups) and the corresponding domain, which will be referred to as activity groups in the following.

**Table 1:** Mean values and standard deviations for the eleven activity groups.

Domain	Activity groups	Mean (M)	Std. dev. (SD)
Home	Home indoor	8.82	3.58
	Home outdoor	2.31	2.32
Occupational	Occupational physical activity	3.26	4.71
Transport	Walking as transportation	2.25	3.22
	Biking as transportation	3.16	3.08
Leisure	Walking/biking	5.07	4.29
	Running/fitness/flexibility training	2.84	3.78
	Ball games	0.38	1.15
	Gymnastics/dance	0.34	1.15
	Water/outdoor activities	0.80	1.81
	Other sport/street activities	0.24	0.99

The results are presented in days per week. (*N* = 143,794).

The eleven activity groups were inspired by categorisations in other research that often take place/organisation and intensity into consideration. An overview of the construction of the eleven activity groups is presented in a supplementary file [see Supplementary File S2].

To ensure the same measurement scale across all activities, the variables were recoded so that the measurement categories for each variable were similar and were presented as an average of the number of days per week the respondents had reported performing the activity. Participants reporting 5 days or more per week were categorised as 6 days a week, 3–4 days a week were categorised as 3.5 days a week, 1–2 days a week were categorised as 1.5 days a week, less than 1 day a week was categorised as 0.5, while ‘never’ and ‘less than once a month’ were categorised as 0. Some of the activities that are summed up in this study are performed quite frequently (e.g., cooking, doing laundry, gardening, walking, biking, etc.), and for that reason the frequency of participation in some categories (e.g., home indoor) exceeds seven times a week on average.

### 2.3 Independent variables

Information about characteristics of social background (gender, age, educational level, occupation, income, origin and health status or disabilities) was also collected in the survey or provided by Statistics Denmark. These social background variables were used as independent variables in the analysis. Furthermore, the eleven activity groups described in the dependent variables section were also used as independent variables in the analysis to examine cumulative and compensatory effects within and across the eleven activity groups that represent the four life domains. Table 2 presents the social background variables.

**Table 2:** The distribution (in per cent) of the respondents according to the social background variables included.

Social background	Percentage (%)
Gender: Male	45.1%
Gender: Female	54.9%
Age: Young (15–29 years)	15.6%
Age: Adult (30–59 years)	46.2%
Age: Elderly (60+ years)	28.2%
Education: Primary	18.6%
Education: Secondary	42.1%
Education: Tertiary	39.3%
Occupation: In education	9.0%
Occupation: In occupation	56.8%
Occupation: Outside labour market	34.2%
Income: Low (€27,000 or less)	18.7%
Income: Medium (€27,000–42,000)	35.8%
Income: High (€42,000 and more)	45.4%
Origin: Danish	93.1%
Origin: Other Western	3.3%
Origin: Non-western	3.6%
Health status or disability: (‘yes’ selected)	
Chronic illness	20.5%
Mental disability	7.0%
Physical disability	8.0%

(*N* = 143,794).

Gender and age data were obtained from the respondents’ social security number through Statistics Denmark. Age was collected as a continuous variable and converted to an ordinal variable with three categories—15–29, 30–59 or 60 + years—to describe different life phases for *young, adult* and *elderly* individuals. Education, occupation, income and origin were classified using data from Statistics Denmark. The education variable was divided into three levels of education: *primary, secondary* or *tertiary* education. Occupation was grouped into three categories: *in occupation, in education* or *outside the labour market*. For those outside the labour market, the majority are pensioners. Income was categorised into three categories as annual equivalised disposable income based on amounts in Danish kroner and converted into Euros: *low* (€27,000 or below), *medium* (€27,000–42,000) or *high* (€42,000 and more) income. The origin variable was categorised into three groups: *Danish origin, other Western origin* and *non-Western origin*. Regarding health status or disabilities, we included three types: *chronic illness* (e.g., allergy, asthma, bronchitis, epilepsy, arthritis), *mental disorder* (e.g., ADHD, ADD, anxiety, autism, depression) or *physical disability* (e.g., amputation, cerebral palsy, cystic fibrosis). The questions regarding health status or disabilities were asked separately with the option to respond either 0 = no (not selected) or 1 = yes (selected).

### 2.4 Data analysis

To analyse this data statistically, a multiple linear regression analysis was performed to examine the two research questions. To answer RQ1, the association and the strength of the coefficients were studied among the eleven activity groups representing the four domains. To address RQ2, the social background variables were studied to examine the association between the participants’ social background and their frequency of participation in the eleven activity groups. The multiple linear regression analysis was conducted as a full model with all variables included the analysis. Checks regarding violations of the assumptions of multiple linear regression analysis (absence of influential outliers, linearity, absence of strong multicollinearity and homogeneity of variance) were performed, and no violations were identified.

## 3 Results

The presentation of the results from the multiple linear regression analysis (Table 3) is separated into two sections, corresponding to the research questions. First, the results of the analysis of the 11 activity groups within the four domains. Next, the analysis of the social background variables. Both sections describe the associations to the frequency of PA within and across the 11 activity groups. Nearly all associations among the variables show statistical significance due to the large sample size of our data. Therefore, the presentation of the results does not cover all statistically significant results but focuses on key results based on the magnitude of the regression coefficients (i.e., unstandardized beta coefficients) from the multiple linear regression analysis.

**Table 3:** Results from the multiple linear regression analysis with unstandardised beta coefficients of the eleven activity groups (dependent/independent) and the selected social background variables (*N* = 143,794).

Activity groups/social background	Home indoor	Home outdoor	Occupational PA^a^	Walking as transportation	Biking as transportation	Walking/biking	Running/fitness/ flexibility training	Ball games	Gymnastics/dance	Water/outdoor activities	Other sport/street activities
(Constant)	5.333***	0.882***	6.042***	1.772***	2.288***	−0.369***	2.261***	0.499***	−0.275***	−0.206***	0.226***
Activity group											
Home indoor		0.105***	0.033***	0.006***	0.052***	0.128***	0.054***	0.004***	0.001	0.002	−0.002**
Home outdoor	0.241***		0.158***	−0.037***	−0.039***	0.167***	−0.054***	−0.005***	0.013***	0.107***	0.017***
Occupational PAa	0.021***	0.043***		0.017***	0.050***	−0.010***	−0.025***	0.003***	0.007***	0.012***	0.005***
Walking as transportation	0.006***	−0.017***	0.030***		0.051***	0.232***	0.054***	0.011***	−0.001	0.005***	−0.002**
Biking as transportation	0.062***	−0.020***	0.097***	0.056***		0.332***	0.017***	0.002***	0.012***	0.010***	−0.003**
Walking/biking	0.086***	0.049***	−0.011***	0.145***	0.187***		0.123***	0.001	0.006***	0.059***	0.003***
Running/fitness/flexibility training	0.044***	−0.019***	−0.033***	0.041***	0.012***	0.149***		0.021***	0.047***	0.036***	0.020***
Ball games	0.036***	−0.020***	0.046***	0.083***	0.016***	0.005	0.222***		0.084***	0.061***	0.125***
Gymnastics/dance	0.011	0.047***	0.095***	−0.001	0.083***	0.072***	0.470***	0.081***		0.093***	0.067***
Water/outdoor activities	0.008	0.158***	0.067***	0.016***	0.027***	0.293***	0.147***	0.024***	0.038***		0.090***
Other sport/street activities	−0.027**	0.082***	0.088***	−0.026**	−0.026**	0.046***	0.269***	0.165***	0.091***	0.300***	
Social background											
Gender: Male (ref.)											
Gender: Female	1.436***	−0.777***	−0.058***	−0.029***	−0.045***	0.320***	0.026*	−0.181***	0.144***	−0.037***	0.005
Age: young (15–29 years) (ref.)											
Age: Adult (30–59 years)	1.274***	0.520***	−0.815***	−0.508***	−0.717***	1.008***	−1.334***	−0.314***	−0.009	0.042*	−0.149***
Age: Elderly (60+ years)	0.338***	1.140***	−1.643***	−0.527***	−0.380***	1.381***	−2.439***	−0.304***	0.161***	0.162***	−0.226***
Education: Primary (ref.)											
Education: Secondary	0.284***	−0.018	−0.245***	0.065***	−0.139***	0.142***	0.348***	−0.103***	−0.086***	0.040**	−0.067***
Education: Tertiary	0.351***	−0.148***	−1.826***	0.693***	−0.015	−0.215***	0.877***	−0.096***	−0.099***	0.163***	−0.075***
Occupation: In occupation (ref.)											
Occupation: In education	−1.270***	0.044	−3.337***	1.409***	1.144**	−0.596***	0.249***	0.477***	0.293***	−0.046*	0.102***
Occupation: Outside labour market	0.066***	0.349***	−4.707***	−0.623***	0.413***	0.209***	−0.099**	0.078***	0.155***	0.063***	−0.005
Income: Low (€27,000 or less) (ref.)											
Income: Medium (€27,000–42,000)	−0.244***	0.321***	0.424***	−0.289***	−0.457***	0.355***	0.015	0.159***	0.047***	−0.053***	−0.023***
Income: High (€42,000 and more)	−0.514***	0.408***	−0.413***	−0.323***	−0.700***	0.686***	0.223***	0.227***	0.040***	−0.024	−0.023**
Origin: Danish (ref.)											
Origin: Other Western	0.087	−0.211***	0.274***	0.205***	0.370***	−0.484***	0.397***	−0.032*	0.156***	0.139***	0.036**
Origin: Non-western	0.030	−0.437***	0.535***	−0.355***	0.619***	−0.834***	0.482***	0.075***	0.300***	−0.085**	−0.023**
Health status or disability (ref. not selected):											
Chronic illness	−0.143***	−0.104***	−0.001	−0.265***	0.029	−0.110***	−0.080**	−0.047***	0.006	−0.008	0.017**
Mental disability	−0.379***	−0.105***	−0.013	−0.409***	0.114***	0.110***	0.042	−0.156***	−0.037**	0.035	0.024*
Physical disability	−0.163***	−0.135***	0.172***	−0.233***	−0.270***	−0.293***	0.012	−0.095***	−0.053***	0.128***	0.016
Adjusted R2	0.236	0.206	0.275	0.122	0.137	0.207	0.164	0.125	0.095	0.110	0.103

* *P* < 0.05; **P < 0.01; ***P < 0.001. ^a^PA, physical activity.

### 3.1 Home domain

#### 3.1.1 Home indoor

The frequency of home indoor activities is significantly positively associated with nine activity groups, most notably with walking/biking (b = 0.128) and home outdoor activities (b = 0.105). Home indoor activities are only significantly negatively associated with other sports/street activities, but with a very low regression coefficient (b = −0.002).

#### 3.1.2 Home outdoor

As for the frequency of the home outdoor activities, six activity groups are significantly positively associated. Home indoor (b = 0.241), occupational PA (b = 0.158), walking/biking (b = 0.167) and water/outdoor activities (b = 0.107) are the most notable. Home outdoor are significantly negatively associated with four activity groups, but with low regression coefficients. These activity groups are running/fitness/flexibility training (b = −0.054), biking as transportation (b = −0.039), walking as transportation (b = −0.037) and ball games (b = −0.005).

### 3.2 Occupational domain

#### 3.2.1 Occupational Pa

Generally, the occupational domain shows very low regression coefficients. Eight of the activity groups are positively significantly associated with occupational PA (b = 0.003–0.043). Home outdoor and biking as transportation show the highest regression coefficients. Occupational PA is significantly negatively associated with running/fitness/ flexibility training (b = −0.025) and walking/biking (b = −0.010), but with low regression coefficients.

### 3.3 Transportation domain

#### 3.3.1 Walking as transportation

Seven of the activity groups are significantly positively associated with walking as transportation, the most notable ones being walking/biking (b = 0.232). Three activity groups are significantly negatively associated with walking as transportation, but with low regression coefficient. These are home outdoor (b = −0.017), other sports/street activities (b = −0.002) and gymnastics/dance (b = −0.001).

#### 3.3.2 Biking as transportation

Eight of the activity groups are significantly positively associated with biking as transportation. Walking/biking (b = 0.332) is the most notable in this case. Home outdoor (b = −0.020) and other sports/street activities (b = −0.003) are significantly negatively associated with biking as transportation, also with low regression coefficients.

### 3.4 Leisure domain

#### 3.4.1 Walking/biking

The frequency of walking/biking activities is significantly positively associated with nine activity groups, particularly biking as transportation (b = 0.187), walking as transportation (b = 0.145) and running/fitness/flexibility training (b = 0.123). Only occupational PA is significantly negatively associated with walking/biking activities (b = −0.011).

#### 3.4.2 Running/fitness/flexibility training

The frequency of running/fitness/flexibility training is significantly positively associated with eight activity groups, particularly walking/biking (b = 0.149). Two activity groups are significantly negatively associated with running/fitness/flexibility training: occupational PA (b = −0.033) and home outdoor (b = −0.019).

#### 3.4.3 Ball game activities

The frequency of ball games is significantly positively associated with nine activity groups, most notably running/fitness/flexibility training (b = 0.222) and other sport/street activities (b = 0.125). Ball games are significantly negatively associated with home outdoor activities (b = −0.020).

#### 3.4.4 Gymnastics/dance

The frequency of gymnastics/dance activities is significantly positively associated with nine activity groups, particularly running/fitness/flexibility training (b = 0.470).

#### 3.4.5 Water/outdoor activities

The frequency of water/outdoor activities is significantly positively associated with all ten activity groups, most notably walking/biking (b = 0.293), home outdoor (b = 0.158) and running/fitness/flexibility training (b = 0.147)

#### 3.4.6 Others sport/street activities

The frequency of other sport/street activities is significantly positively associated with seven activity groups, particularly water/outdoor activities (b = 0.300), running/fitness/flexibility training (b = 0.269) and ball games (b = 0.165). Three activity groups are significantly negatively associated with other sport/street activities. These are biking as transportation (b = −0.045), walking as transportation (b = −0.029) and home indoor (b = −0.027).

### 3.5 Social background

#### 3.5.1 Gender

The frequency of women's PA is positively associated with five activity groups, most notably with home indoor (b = 1.436) and the leisure activities walking/biking (b = 0.320) and gymnastics/dance (b = 0.144) compared to men's PA. A significant negative association for women is observable in six activity groups, most notably in home outdoor (b = −0.777) and in ball games (b = −0.165) compared with men's PA.

#### 3.5.2 Age

PA at home outdoor (b_adult_ = 0.520 and b_elderly_ = 1.140), walking/biking (b_adult_ = 1.008 and b_elderly_ = 1.381) and water/outdoor activities (b_adult_ = 0.042 and b_elderly_ = 0.162) increase with age. The opposite holds for occupational PA (b_adult_ = −0.815 and b_elderly_ = −1.643), walking as transportation (b_adult_ = −0.508 and b_elderly_ = −0.527) and the leisure activities running/fitness/flexibility training (b_adult_ = −1.334 and b_elderly_ = −2.439) and other/street activities (b_adult_ = −0.149 and b_elderly_ = −0.226).

#### 3.5.3 Education

With increasing educational level, PA at home indoor (b_secondary_ = 0.284 and b_tertiary_ = 0.351), walking as transportation (b_secondary_ = 0.065 and b_tertiary_ = 0.693), running/fitness/flexibility training (b_secondary_ = 0.348 and b_tertiary_ = 0.877) and water/outdoor activities (b_tertiary_ = 0.040 and b_tertiary_ = 0.163) become more prevalent. In contrast, the frequency of PA at home outdoor (b_secondary_ = −0.018 and b_tertiary_ = −0.148) and occupational PA (b_secondary_ = −0.245 and b_tertiary_ = −1.826) become less prevalent with increasing educational level.

#### 3.5.4 Occupation

Compared to individuals in occupation, the frequency of PA for individuals in education and individuals outside the labour market is positively associated with around half of the activities, in particular PA at home outdoor (b_outside labour market_ = 0.349), biking as transportation (b_in education_ = 1.144 and b_outside labour market_ = 0.413), ball games (b_in education_ = 0.477) and gymnastics/dance (b_in education_ = 0.293 and b_outside labour market_ = 0.155). Specifically for individuals in education, a significant positive association is shown with walking as transportation (b_in education_ = 1.409) and running/fitness/flexibility training (b_in education_ = 0.249) compared to individuals in occupation. For individuals outside the labour market, the frequency of PA is also significantly positively associated with walking/biking during leisure (b_outside labour market_ = 0.209). The opposite is prevalent for occupational PA (b_in education_ = −3.337 and b_outside labour market_ = −4.707), which is significantly negatively associated.

#### 3.5.5 Income

With increasing income, PA at home outdoor (b_medium_ = 0.321 and b_high_ = 0.413) and the leisure activities walking/biking (b_medium_ = 0.355 and b_high_ = 0.686), running/fitness/flexibility training (b_medium_ = 0.015 and b_high_ = 0.223) and ball games (b_medium_ = 0.159 and b_high_ = 0.227) become more prevalent. The opposite is the case for PA at home indoor (b_medium_ = −0.244 and b_high_ = −0.514), walking as transportation (b_medium_ = −0.289 and b_high_ = −0.323) and biking as transportation (b_medium_ = −0.457 and b_high_ = −0.700) which show significantly negative associations.

#### 3.5.6 Origin

The frequency of PA in both groups of foreign origin, other Western and non-Western origin, are significantly positively associated with occupational PA (b_other western_ = 0.274 and b_non−western_ = 0.545), biking as transportation (b_other western_ = 0.370 and b_non−western_ = 0.619) and the leisure activities running/fitness/flexibility training (b_other western_ = 0.397 and b_non−western_ = 0.482) and gymnastics/dance (b_other western_ = 0.156 and b_non−western_ = 0.300), compared to individuals of Danish origin. A negative association is seen in PA at home outdoor (b_other western_ = −0.211 and b_non−western_ = −0.437) and in walking/biking during leisure (b_other western_ = −0.484 and b_non−western_ = −0.834).

#### 3.5.7 Health status and disabilities

Regarding health status and disabilities, we find more negative than positive associations. In particular, the frequency of PA at home indoor (b_chronic_ = −0.143, b_mental_ = −0.379, and b_physical_ = −0.163), home outdoor (b_chronic_ = −0.104, b_mental_ = −0.105 and b_physical_ = −0.135) and walking as transportation (b_chronic_ = −0.265, b_mental_ = −0.409 and b_physical_ = −0.233) is significantly negatively associated for individuals with a chronic illness, individuals with a mental disorder and individuals with a physical disability, compared to those without any health conditions or disabilities.

## 4 Discussion

In the following, we discuss the findings from this study in relation to results from previous studies. First, we discuss our finding that PA generally seems to lead to more PA in different life domains. Secondly, we discuss the variations in social background of PA participation within and across life domains. Finally, we reflect on the implications of our findings for research on PA in life domains and present the limitations of this study.

### 4.1 Physical activity leads to more physical activity within and across life domains

Our analysis finds mainly positive associations between the frequency of participation in all eleven included activity groups, which indicates a cumulative effect and shows that PA in one activity group is most often positively associated with PA in another activity group both within and across domains. There are rare exceptions to this, all of which hold low regression coefficients.

Some studies similarly find positive associations and a cumulative effect (18–20). One study examined associations between PA in the home, transportation and occupational domains and found that individuals who are active during transportation were less likely to exhibit sedentary leisure activities compared with individuals who are not active during transportation (18). Another study identified cumulative effects between PA in the transportation and leisure domains (19). In sum, the studies cited have only examined PA in selected domains and have mainly identified cumulative effects between the transport and leisure domains.

Other studies report contradictory findings to our main finding that PA in one domain is positively associated with PA in other domains. These studies find that PA in one domain compensates for sedentary behaviour in another domain (21, 22), one study showing that leisure activities are connected to profession in the sense that directors/managers/office workers are more physically active in leisure activities compared with skilled workers. This indicates a compensatory relationship regarding PA between the work and leisure domains (21).

Our study contributes to the literature by providing information about associations among PA levels in all four domains. We have not been able to identify other studies that include all domains, nor studies that investigate associations among activity groups and social background variables. With this approach, we find mainly positive associations among PA in all four domains, which indicates a primarily cumulative effect in PA across life domains, but there are also examples in our data of compensatory effects, although they are rare, and the effect sizes are modest. Thus, our study seems to indicate that people tend to have either an overall physically active or non-active lifestyle, and that being frequently active in one domain is generally not a barrier for being physically active in other domains.

It is worth noting that differences in results between our study and some of the cited studies may be attributed to variations in how PA is measured. Some studies have used different methods to assess PA, such as MET scores, minutes per week (20–22) or accelerometer data (19). In addition, only in some of the studies does the study population constitute a representative sample of the adult population. Some studies only include individuals who are active in the labour market (21) and some focus on a specific group, such as adolescents (20). The categorisation and inclusion of activity domains also vary: some studies focus on the work, transport and leisure domains (20), others on leisure and transport domains (19). Furthermore, the operationalisation of PA also varies somewhat in the studies cited. In this context, it is worth mentioning that some studies focused on sedentary time rather than PA (20). The income level of the population and the country in which the study is conducted must also be considered, particularly when comparing low-, middle-, and high-income countries. Denmark, a high-income country, may show different results compared to studies from countries such as Poland (21) and Brazil (18) due to differences in income and socioeconomic status.

### 4.2 Variations in the association between social background and physical activity

Most existing studies paint a relatively uniform picture of who is more likely to engage in PA. For instance, men have been found to be more physically active than women in leisure activities (14, 18). Young people have been found to be more physically active than the elderly in leisure activities (23). People with a high income have been found to be more physically active in the leisure domain than people with a lower income (12). Also, people in occupation have been found to be more physically active in the leisure domain than people outside the labour market (23).

Our study nuances these general findings, thereby illustrating the usefulness of the approach in our study which differentiates among eleven different activity groups and examine the effects of social background variables in relation to each of these. Understanding the different ways in which people incorporate PA into different domains of life contributes to a comprehensive understanding of behavioural patterns (4). Although we were able to identify a few studies that examined the associations between social background variables and PA in some domains (21, 22), we did not find any studies that simultaneously examined the associations between social background and PA in all four domains and/or subdivided into activity types within each of the domains.

In our study, the activity groups walking/biking and running/fitness/flexibility training show the overall highest regression coefficient within and across the activity groups. This reflects that these activity groups are relatively often practiced in combination with other activity groups by many of the respondents, which is clearly illustrated by the cumulative effect. In the Danish population, the top five leisure activities practiced every week are walking, fitness (both strength and cardio), biking, running and yoga (which belong to the activity group categorised as ‘flexibility training’) (23). This clarifies the popularity of these activity groups among Danes and explains the high coefficients in our study.

A noteworthy finding is the differences found in the effects of education level and income within the home domain. For home indoor activities, we find that the frequency of PA increases with educational level but decreases with income. For home outdoor activities, we find the opposite effects. This provides interesting nuances to the general finding in previous studies that higher education and general high socioeconomic status (SES) are often associated with less PA in the home domain (12, 17). A different pattern emerges when home activities are subdivided. For example, people with medium to high income tend to be more physically active in outdoor home activities compared with people with low income. However, people with medium to high income are less physically active in indoor home activities compared with people with low income. Perhaps people with medium to high income have invested in cleaning help, but they enjoy and relax with projects in the garden. This is relevant for future research regarding PA in domains where nuances and exceptions may also apply.

We find that participants of a young age, primary education, low and medium income, other Western and non-Western origin are more frequently physically active in the occupation domain. This is consistent with previous research indicating that occupational PA is more frequent among individuals with lower SES (17). Our analysis also shows that participants with health disabilities or issues are more frequently physically active in the occupation domain compared with participants without health disabilities or issues, which may indicate that the physically active work may have led to physical deterioration.

Generally, we find that increasing age and higher income are associated with less frequent physical activity in the transportation domain. However, a difference observed in PA in the transportation domain is that people not in occupation are more frequently walking as transportation than people in occupation, whereas the opposite holds for biking as transportation. Other studies have not subdivided active transportation and therefore do not find the same nuances among these groups as our study.

An overall and important result of our study is the finding that the regression coefficients, and therefore the explanatory power of independent variables of our analysis, are greater among the social background variables and PA participation in the different domains than among the eleven activity groups. This indicates that social background variables are more strongly associated with the frequency of PA than with the activity groups. Therefore, it appears that social background plays an important role for the frequency of PA in different activity groups and domains.

### 4.3 Implications for research on PA in life domains

At least two implications from our study in relation to research on PA are relevant to mention. First, our study shows that social background is a relevant factor to include in the analysis of PA within and across domains. This is justified by our finding that social background is an important determinant of PA, and by the variance of the effects of the social background variables both within and among domains.

This leads to the second important implication of our study: when examining PA, it is worth subdividing it into activity groups within domains. The many differences identified in effect sizes of the independent variables among these activity groups—both within and across domains—justify the need for this nuanced approach. We have not been able to identify such an approach in other studies to an extent that is comparable to our study.

### 4.4 Limitations

For this study, some limitations regarding the analysis are worth mentioning. To get an even clearer picture of the associations among the activity groups and in relation to social background effects, the activity groups might have been subdivided further. However, we chose to limit the number of activity groups to conduct the analyses with a suitable number of dependent variables.

Another limitation of the study is that the data presents the number of days per week on which the respondents are active, and therefore it does not show the intensity or duration of the activity. The use of other measures for examining physical activity than days per week, e.g., intensity or duration, makes it challenging to compare studies of physical activity. However, in that respect our study also brings forth an alternative way of measuring PA.

## 5 Conclusions

The article examined the association between the frequency of PA and social background across home, occupational, transportation and leisure domains. This was achieved by dividing PA into eleven activity groups within and across the four life domains and conducting a multiple linear regressions analysis with seven additional social background variables to study associations between PA levels within the eleven activity groups and social background variables.

We found that the associations among the activity groups indicate that PA in one activity group is most often positively associated with PA in another activity group both within and across domains, i.e., predominantly cumulative effects. However, social background factors play an even greater importance in explaining differences in individuals’ participation in PA within and across domains. We find different effects of social background variables among the eleven different activity groups, which demonstrates that the effects of social background are not uniform across all forms of physical activity. Most existing studies paint a relatively uniform picture of who is more likely to engage in PA. Our study nuances these general findings, thereby illustrating the usefulness of the approach in our study which differentiates among eleven different activity groups and examine the effects of social background variables in relation to each of these.

In relation to RQ1, the study finds that participation in PA within and across life domains is predominantly characterised by positive, cumulative associations between different activity types. With regard to RQ2, the study demonstrates that social background factors play a significant and differentiated role in explaining variation in PA participation, with their influence varying considerably across specific activity groups.

## Data Availability

Due to data protection regulation and the sensitive nature of parts of the data collected in the survey study, the data is not publicly available. For any queries regarding the data, please contact Karsten Elmose-Østerlund at kosterlund@health.sdu.dk.
