# Supplementary material for: The association between the frequency of physical activity and social background within and across home, occupational, transportation and leisure domains
Source: Front Sports Act Living. 2025 Aug 12;7:1609485. doi: 10.3389/fspor.2025.1609485 (PMC12378642; doi:10.3389/fspor.2025.1609485)
Supplement: Supplementary file 1 [file Datasheet1.pdf]

QUESTIONNAIRE FOR ’MOVING DENMARK’

[INTRODUCTION PAGE/INSTRUCTION FOR ALL RESPONDENTS]

Thank you for your part in creating ‘Moving Denmark’!

The questionnaire is divided into six parts:

1) Practical work at home
2) Physical activity at work or during your studies
3) Physical activity such as transport (e.g. walking, biking and running)
4) Physical activity during leisure time
5) Opportunities for physical activity in the area where you live
6) Questions about you (your household, your working life and your health)

COVID-19/corona may have affected how and how often you are physically active. Therefore, we ask you to answer the questions based on an overall assessment of the past 12 months. At the end of the questionnaire, we ask how COVID-19/corona has affected your physical activity.

It takes approximately 15 minutes to answer the questionnaire.

Use the buttons at the bottom of each page to navigate back and forth in the questionnaire. You have the opportunity to take a break from filling in the questionnaire. Your answers will be saved, and you will be able to return to the questionnaire later to complete it by clicking on the link in the invitation letter. At the end of the questionnaire, it is possible to print your own answers.

Click ‘Næste’ (Next) to begin answering the questionnaire.

PART 1: PRACTICAL WORK AT HOME

1. How often do you perform the following types of practical work at home (indoors and outdoors)?
(please tick one box in each line)

	**5 days a week or more**	**3-4 days a week**	**1-2 days a week**	**Less than 1 day a week**	**Never**
Cook, clear the table and do the dishes	❑	❑	❑	❑	❑
Clean	❑	❑	❑	❑	❑
Do the laundry	❑	❑	❑	❑	❑
Garden (e.g. mow the lawn, sweep, weed, rake leaves, dig)	❑	❑	❑	❑	❑
Other practical work (e.g. maintenance and repair of home, car, bike, etc. and other ‘do-it-yourself’ projects)	❑	❑	❑	❑	❑

PART 2: PHYSICAL ACTIVITY AT WORK OR DURING YOUR STUDIES

The following question deals with your main occupation. If you work or study, you will afterwards be asked a question about the physical activity that is part of your work or studies.

2. What is your main occupation at the moment?
(please tick only one box)

Undergoing education/going to school (incl. apprentices and trainees)	❑
Unskilled job (job that does not require an education), e.g. job in a factory, cleaning, childcare assistant, etc.	❑
Vocational job, e.g. job in an office, craftsman, social and health care worker, etc.	❑
Job following a short-cycle higher education, e.g. computer specialist, marketing economist, pharmaconomist, electrician, laboratory technician, etc.	❑
Job following a medium-cycle higher education, e.g. nurse, midwife, physiotherapist/occupational therapist, pre-school, primary school and lower secondary school teachers, journalist, certificated engineer, social worker, etc.	❑
Job following a long-cycle higher education, e.g. doctor, upper secondary school teacher, lawyer, civil engineer, researcher, etc.	❑
Self-employed (you have your own company or are co-owner of a company)	❑
Unemployed (receive unemployment benefit/labour market benefit from unemployment insurance fund)	❑
Unemployed on social security benefits	❑
Disability pensioner	❑
Retirement pensioner, early retirement pensioner	❑
On leave of absence from work/course of study (e.g. maternity leave, nursing leave, sick leave)	❑
On leave of absence from unemployment benefits/social security benefits (e.g. maternity leave, nursing leave, sick leave)	❑
Non-working	❑
Other (please specify)	❑

[SHOWN TO RESPONDENTS WHO WORK OR ARE UNDERGOING EDUCATION (BASED ON QUESTION 2)]

3. How often is your work or studies characterised by?
(please tick one box in each line)

	**5 days a week or more**	**3-4 days a week**	**1-2 days a week**	**Less than 1 day a week**	**Never**
Tasks that do not require physical exertion (e.g. sedentary or stationary)	❑	❑	❑	❑	❑
Tasks with light physical exertion (e.g. repeated standing movements or walking)	❑	❑	❑	❑	❑
Tasks with moderate physical exertion (e.g. repeated lifting or use of tools)	❑	❑	❑	❑	❑
Tasks with heavy physical exertion (e.g. repeated heavy lifting)	❑	❑	❑	❑	❑

PART 3: PHYSICAL ACTIVITY AS TRANSPORT

[SHOWN TO RESPONDENTS WHO WORK OR ARE UNDERGOING EDUCATION (BASED ON QUESTION 2)]

4. How often do you use the following modes of transport on the way <u>to and from your work or place of study</u> (the entire trip or large parts of it)?
(please tick one box in each line)

	**5 days a week or more**	**3-4 days a week**	**1-2 days a week**	**Less than 1 day a week**	**Never**
Biking (incl. electric bicycle)	❑	❑	❑	❑	❑
Walking	❑	❑	❑	❑	❑
Running (incl. roller skates, skateboards and scooters)	❑	❑	❑	❑	❑

5. How often do you use the following modes of transport <u>to and from shopping, institutions, leisure activities, visiting friends and family and the like</u> (the entire trip or large parts of it)?
(please tick one box in each line)

	**5 days a week or more**	**3-4 days a week**	**1-2 days a week**	**Less than 1 day a week**	**Never**
Biking (incl. electric bicycle)	❑	❑	❑	❑	❑
Walking	❑	❑	❑	❑	❑
Running (incl. roller skates, skateboards and scooters)	❑	❑	❑	❑	❑

[SHOWN TO RESPONDENTS WHO HAVE TICKED AT LEAST ONE BOX FOR 1-2 DAYS A WEEK, 3-4 DAYS A WEEK OR 5 DAYS A WEEK OR MORE (QUESTIONS 4 AND 5)]
The next question deals with the reasons why you walk, bike or run as transport.

6. How much do you agree or disagree with the following statements about why you walk, bike or run as transport?
(please tick one box in each line)

**I walk, bike or run…**	**Completely agree**	**Partly agree**	**Neither nor**	**Partly disagree**	**Completely disagree**
To maintain or improve my health (e.g. physical fitness)	❑	❑	❑	❑	❑
To spend time with others	❑	❑	❑	❑	❑
To do something nice for myself	❑	❑	❑	❑	❑
To maintain or improve my appearance	❑	❑	❑	❑	❑
Because others in my social circle encourage me to do so	❑	❑	❑	❑	❑
Because I like to walk, bike or run	❑	❑	❑	❑	❑
Because I feel good afterwards	❑	❑	❑	❑	❑
Because it is the cheapest mode of transport	❑	❑	❑	❑	❑
Because it is the fastest or easiest mode of transport	❑	❑	❑	❑	❑
Because it is good for the environment and the climate	❑	❑	❑	❑	❑

Whether you often, rarely or never bike, walk and run as transport, there may be good reasons why you choose to transport yourself in other ways.

7. What keeps you from walking, biking or running (more often) as transport?
(it is possible to tick multiple boxes)

Nothing – I am not interested in walking, biking or running (more often) when transporting myself	❑
That it takes too long and/or the distances are too great	❑
That I feel unsafe on the road (e.g. due to traffic, lack of pavement, bike path or lighting)	❑
That I often run errands on the way or drive others (e.g. colleagues, children)	❑
That I feel embarrassed when I walk, bike or run	❑
That I do not like to be hot and sweaty	❑
That I do not feel presentable after walking, biking or running	❑
That I lack the necessary skills	❑
That I do not have anyone to walk, bike or run with	❑
That others in my social circle do not encourage me to walk, bike or run	❑
That I am often too exhausted or tired	❑
That I prioritise time at my job or with my family	❑
That I am in need of or have poor equipment (e.g. bike)	❑
That I have a disability or a chronic disease	❑
That I am in bad shape	❑
That I am injured	❑
That I am worried about getting injured	❑
That it is often bad weather (e.g. rain or wind)	❑
That I like driving	❑
Other (please specify)	❑

PART 4: PHYSICAL ACTIVITY DURING LEISURE TIME

The first questions deal with what activities you do in your leisure time and how often. In this connection, you must include exercise and training at the workplace as well as training and playing at home (indoors and outdoors).

8. Which of the following physical activities have you performed within the last 12 months?
(please tick one box in each line)

	Yes	No
Walking and hiking (not as transport)	❑	❑
Running (not as transport)	❑	❑
Biking (not as transport)	❑	❑
Fitness and physical training (e.g. strength exercises/weight training, cardio workout, team training)	❑	❑
Mental training and flexibility/stability training (e.g. yoga, Pilates, meditation/mindfulness)	❑	❑
Team ball games (e.g. football, handball, volleyball, beach volley, floorball)	❑	❑
Other ball games (e.g. golf, badminton, table tennis, tennis, squash)	❑	❑
Gymnastics (e.g. spring gymnastics, rhythmic gymnastics, apparatus work, keep-fit exercises)	❑	❑
Dance (e.g. partner dance, fitness dance, modern dance, creative dance, street dance)	❑	❑
Activities in water (e.g. swimming, diving, winter swimming)	❑	❑
Activities on the water (e.g. canoeing/ kayaking, rowing, sailing, surfing, stand up paddle)	❑	❑
Outdoor activities (e.g. outdoor life, fishing, hunting, scouts, role-playing)	❑	❑
Rolling and street activities/street sports (e.g. roller skating, scooters, skateboarding, parkour, street basketball)	❑	❑
Rehabilitation (e.g. in hospital, at municipal level, private, at home)	❑	❑
Physically active games (e.g. playing with children, croquet, rounders, dodge ball, hide and seek)	❑	❑
Other sporting activities (e.g. athletics, bowling, martial arts, pétanque, horseback riding, roller skiing, skiing, shooting, triathlon)	❑	❑
Other physically active leisure activities (e.g. handicrafts, arts and crafts, food and gastronomy, theatre, music)	❑	❑

[SHOWN TO RESPONDENTS WHO HAVE CHOSEN AT LEAST ONE TYPE OF ACTIVITY (IN QUESTION 8). ONLY THE SELECTED TYPES OF ACTIVITY ARE SHOWN]

In the previous question, you ticked selected types of activity. In this question, we ask that for each of the selected types of activity you tick the activities you have performed within the last 12 months.

9. Which activities have you performed within the last 12 months?
(it is possible to tick multiple boxes)

**Walking and hiking (not as transport)**	
Walking at a fast pace (e.g. Power Walking, Speed Walking)	❑
Walking/hiking at a moderate pace	❑
Walking with a dog	❑
Other types of walking and hiking (please specify)	❑

**Running (not as transport)**	
Jogging	❑
Orienteering	❑
Cross-country running (e.g. trail running)	❑
Other types of running (please specify)	❑

**Biking (not as transport)**	
Road cycling (on a racing bike)	❑
Mountain biking	❑
Biking (on ‘everyday bike’)	❑
Other forms of biking (please specify)	❑

**Fitness and physical exercise**	
Strength exercises/weight training (e.g. with weights, on machines, functional training)	❑
Cardio workout (e.g. on a treadmill, exercise bike or rowing machine)	❑
Aerobics/step aerobics	❑
Crossfit	❑
Spinning	❑
Other forms of team training (e.g. body pump, body attack, body balance, circuit training, TRX, tighten up, stretching, fitness boxing)	❑
Other forms of fitness and physical training (please specify)	❑

**Mental training and flexibility/stability training**	
Yoga	❑
Pilates	❑
Meditation/mindfulness	❑
Tai chi, qi gong	❑
Other forms of mental training and flexibility/stability training (please specify)	❑

**Team ball games**	
Basketball	❑
Football	❑
Hockey/floorball	❑
Handball	❑
Volleyball/beach volley	❑
Other team ball games (please specify)	❑

**Other ball games**	
Badminton	❑
Table tennis	❑
Golf	❑
Padel	❑
Squash	❑
Tennis	❑
Other ball games (please specify)	❑

**Gymnastics**	
Spring gymnastics and/or rhythmic gymnastics (e.g. team gym, tumbling, grand prix, acro)	❑
Apparatus work (e.g. parallel bars, floor, rings, vaulting horse)	❑
Keep-fit exercises	❑
Trampoline	❑
Other forms of gymnastics (please specify)	❑

**Dance**	
Partner dance (e.g. folk dance, ballroom, sports dance, salsa, tango, swing)	❑
Ballet, flamenco, modern dance and the like	❑
Fitness dance (e.g. Zumba)	❑
Creative dance (e.g. project-oriented dance with professional dancer)	❑
Street dance (e.g. hip hop, house, breakdance, shuffle, dancehall)	❑
Other types of dancing (please specify)	❑

**Activities in water**	
Swimming in a swimming facility/pool	❑
Open Water Swimming	❑
Diving/free diving/underwater hunting	❑
Pool training (e.g. water aerobics, strength training in water, hot water training)	❑
Winter swimming	❑
Other activities in water (please specify)	❑

**Activities on water**	
Canoe/kayak	❑
Rowing	❑
Sailing	❑
Windsurfing, kitesurfing	❑
Surfing, stand up paddle	❑
Other activities on water (please specify)	❑

**Outdoor activities**	
Outdoor life	❑
Hunting	❑
Fishing	❑
Scouts	❑
Role-playing (outdoors)	❑
Other outdoor activities (please specify)	❑

**Rolling and street activities/street sports**	
Callisthenics, street workout	❑
Scooter, BMX	❑
Parkour, free running, tricking	❑
Roller skating	❑
Skateboard, longboard, carver board	❑
Street basketball, street soccer, panna	❑
Other rolling and street activities/street sports (please specify)	❑

**Rehabilitation**	
Rehabilitation in a hospital	❑
Municipal level rehabilitation	❑
Private rehabilitation (e.g. with a physiotherapist or occupational therapist)	❑
Rehabilitation at home	❑
Other forms of rehabilitation (please specify)	❑

Other sports activities	
Athletics	❑
Billiards, pool, snooker	❑
Bowling, skittles	❑
Archery	❑
Martial arts (e.g. boxing, karate, aikido, taekwondo, judo, wrestling, fencing)	❑
Pétanque	❑
Horseback riding	❑
Roller skiing	❑
Skiing (e.g. alpine skiing, cross-country skiing, snowboarding, telemark)	❑
Shooting	❑
Triathlon/duathlon	❑
Other sports activities (please specify)	❑

Other physically active leisure activities	
Crafts (e.g. handicraft, upholstery, 'do-it-yourself')	❑
Arts and crafts (e.g. ceramics, crochet, knitting, sewing, embroidery)	❑
Food and gastronomy	❑
Theatre and performing arts (e.g. amateur theatre, dilettante, acting)	❑
Musical performance (e.g. playing an instrument, playing in an orchestra, singing in a choir)	❑
Other physically active leisure activities (please specify)	❑

[SHOWN TO RESPONDENTS WHO HAVE CHOSEN AT LEAST ONE TYPE OF ACTIVITY (IN QUESTION 9). ONLY THE SELECTED ACTIVITIES ARE SHOWN]

In the previous question, you ticked selected activities. In this question, we ask that for each of the selected activities you indicate how often you have performed them within the last 12 months.

10. How often have you performed the following activities within the last 12 months? If there are large fluctuations in how often you performed the activities during the year, please indicate an average for the period in which you performed the activities.
(please tick one box in each line)

	**5 days a week or more**	**4 days a week**	**3 days a week**	**2 days a week**	**1 day a week**	**1-3 days a month**	**Less often than 1 day a month**
Activity 1	❑	❑	❑	❑	❑	❑	❑
Activity 2	❑	❑	❑	❑	❑	❑	❑
Activity 3	❑	❑	❑	❑	❑	❑	❑
Activity 4	❑	❑	❑	❑	❑	❑	❑
Activity 5	❑	❑	❑	❑	❑	❑	❑

[SHOWN FOR ALL ACTIVITIES WHICH THE RESPONDENTS HAVE CARRIED OUT AT LEAST ONCE A WEEK FOR THE PAST 12 MONTHS (BASED ON THE RESPONSE TO QUESTION 10). ONLY THESE ACTIVITIES ARE SHOWN]

The following questions are about the activities that you – according to your response to the previous question – have performed at least once a week for the past 12 months.

11. With whom have you done the following activities in the past 12 months?
(it is possible to tick multiple boxes)

	**Spouse/partner/boyfriend/girlfriend**	**Child/children**	**Other family (e.g. siblings, parents)**	**Friends/acquaintances**	**Colleagues/fellow students**	**Other people**	**Alone**
Activity 1	❑	❑	❑	❑	❑	❑	❑
Activity 2	❑	❑	❑	❑	❑	❑	❑
Activity 3	❑	❑	❑	❑	❑	❑	❑
Activity 4	❑	❑	❑	❑	❑	❑	❑
Activity 5	❑	❑	❑	❑	❑	❑	❑

[SHOWN FOR ALL ACTIVITIES WHICH THE RESPONDENTS HAVE CARRIED OUT AT LEAST ONCE A WEEK FOR THE PAST 12 MONTHS (BASED ON THE RESPONSE TO QUESTION 10). ONLY THESE ACTIVITIES ARE SHOWN]

12. In what contexts have you performed the following activities in the last 12 months?
(it is possible to tick multiple boxes)

	**Association/club**	**Commercial centre (e.g. fitness centre or dance institute)**	**Evening school**	**Company sports/at the workplace**	**Organised via social media (e.g. Facebook groups)**	**Self-organised/on your own (alone or with others)**	**Other context**
Activity 1	❑	❑	❑	❑	❑	❑	❑
Activity 2	❑	❑	❑	❑	❑	❑	❑
Activity 3	❑	❑	❑	❑	❑	❑	❑
Activity 4	❑	❑	❑	❑	❑	❑	❑
Activity 5	❑	❑	❑	❑	❑	❑	❑

[SHOWN FOR THE TYPES OF ACTIVITY TICKED BY THE RESPONDENTS (IN QUESTION 8). WITH THE ADDITION THAT THEY MUST HAVE SUBSEQUENTLY INDICATED TO HAVE PERFORMED ONE ACTIVITY WITHIN THE CATEGORY AT LEAST ONCE A WEEK FOR THE PAST 12 MONTHS (IN QUESTION 10)]

13. How much do you agree or disagree with the following statements about why you are physically active in/by [ACTIVITY TYPE] ?
(please tick one box in each line)

**I perform the activity…**	**Completely agree**	**Partly agree**	**Neither nor**	**Partly disagree**	**Completely disagree**
To get better at the activity	❑	❑	❑	❑	❑
To maintain or improve my health (e.g. physical fitness)	❑	❑	❑	❑	❑
To spend time with others	❑	❑	❑	❑	❑
To do something nice for myself	❑	❑	❑	❑	❑
To maintain or improve my appearance	❑	❑	❑	❑	❑
Because others in my social circle encourage me to do so	❑	❑	❑	❑	❑
Because I am good at the activity	❑	❑	❑	❑	❑
Because I like the activity	❑	❑	❑	❑	❑
To compete with myself or others	❑	❑	❑	❑	❑
Because the activity fits in well with my everyday life	❑	❑	❑	❑	❑
Because I can participate, even though I do not have much experience with the activity	❑	❑	❑	❑	❑

No matter the amount of physical activities you do in your leisure time, there may be good reasons why you are less active than you want, or why you drop some types of activity.

14. What keeps you from being (more) physically active in your leisure time?
(it is possible to tick multiple boxes)

Nothing – I am not interested in being (more) physically active in my leisure time	❑
That it takes too long	❑
That I feel embarrassed when I am physically active	❑
That I do not like to be hot and sweaty	❑
That I lack the necessary skills	❑
That I am in need of someone to be physically active with	❑
That others in my social circle do not encourage me to be physically active	❑
That I am often too exhausted or tired	❑
That I prioritise time at my job or with my family	❑
That I prioritise other leisure activities	❑
That I lack facilities or suitable places to be physically active	❑
That I am in need of or have poor equipment (e.g. bike)	❑
That I have a disability or a chronic disease	❑
That I am in bad shape	❑
That I am injured	❑
That I am worried about getting injured	❑
That I have not been able to find an activity that suits me	❑
That the activities I would like to do are too expensive	❑
That I am worried about not fitting in	❑
Other (please specify)	❑

16. How often were you physically active <u>in your leisure time</u> when you attended primary/lower secondary school (first 10 years of school)? If there were large fluctuations in how physically active you were, please select the statement that best describes the majority of your school life.
(please tick only one box)

I was usually physically active in my leisure time every day	❑
I was usually physically active in my leisure time several times a week	❑
I was usually physically active in my leisure time once a week or less	❑
I was usually not physically active in my leisure time	❑
Do not know/cannot remember	❑

17. Which of the following people in your immediate social circle are regularly physically active <u>in their leisure time</u>?
(please tick one box in each line)

	**Yes**	**No**	**Not relevant**	**Do not know**
Spouse/partner/boyfriend/girlfriend	❑	❑	❑	❑
One or both of my parents	❑	❑	❑	❑
At least one of my siblings (incl. any ’bonus siblings’)	❑	❑	❑	❑
At least one of my children (incl. any ’bonus children’)	❑	❑	❑	❑
One or more friends	❑	❑	❑	❑

<u>PART 5: OPPORTUNITIES FOR PHYSICAL ACTIVITY</u>
The following questions concern the area you live in and your assessment of the opportunities to be physically active in the area.

18. How much do you agree or disagree with the following statements about the area you live in?
(please tick one box in each line)

	**Completely agree**	**Partly agree**	**Neither nor**	**Partly disagree**	**Completely disagree**	**Do not know/not relevant**
It is possible to go grocery shopping close by (e.g. grocer’s, bakery, supermarket)	❑	❑	❑	❑	❑	❑
There are green areas close by (e.g. park, forest, nature)	❑	❑	❑	❑	❑	❑
There are pavement or bike paths by most of the busy roads in the area	❑	❑	❑	❑	❑	❑
There are several paths in the area	❑	❑	❑	❑	❑	❑
The traffic is hectic in the area	❑	❑	❑	❑	❑	❑
People often walk, run or bike in the area	❑	❑	❑	❑	❑	❑
There are good opportunities for public transport in the area	❑	❑	❑	❑	❑	❑
Moving around the area feels safe	❑	❑	❑	❑	❑	❑
The area is beautiful	❑	❑	❑	❑	❑	❑
There are various organised activities in the area (e.g. in associations, clubs, centres or Facebook groups)	❑	❑	❑	❑	❑	❑

19. How much do you agree or disagree with the following statements about the opportunities for physical activity in the area where you live?
(please tick one box in each line)

**I have good opportunities to be physically active…**	**Completely agree**	**Partly agree**	**Neither nor**	**Partly disagree**	**Completely disagree**	**Do not know/not relevant**
In indoor sports facilities (e.g. sports centres, swimming facilities, gymnasiums)	❑	❑	❑	❑	❑	❑
In outdoor sports facilities (e.g. football fields, tennis courts, riding arenas, golf courses, skating rinks)	❑	❑	❑	❑	❑	❑
In commercial sports facilities (e.g. fitness centres, bowling alleys, dance schools)	❑	❑	❑	❑	❑	❑
In green areas (e.g. park, forest, nature)	❑	❑	❑	❑	❑	❑
On or in water (e.g. lake, river, sea, fjord)	❑	❑	❑	❑	❑	❑
At home (inside or outside)	❑	❑	❑	❑	❑	❑
At my place of study or my workplace (e.g. gym)	❑	❑	❑	❑	❑	❑
On roads, paths and pavements	❑	❑	❑	❑	❑	❑

[ITEM 'AT MY PLACE OF STUDY OR MY WORKPLACE (E.G. GYM)' IS SHOWN TO RESPONDENTS WHO WORK (BASED ON QUESTION 2)'

PART 6: QUESTIONS ABOUT YOU

The last questions concern you, including your household, your working life and your health.

20. Who lives in your household (besides you)?
(it is possible to tick multiple boxes)

Spouse/partner/boyfriend/girlfriend	❑
My child/children (incl. any 'bonus children')	❑
One or both of my parents	❑
My brother/sister/siblings (incl. any 'bonus siblings')	❑
Other family members (e.g. my grandparents)	❑
Other non-family members (e.g. my friends, fellow students)	❑
No one, I live alone	❑

[SHOWN FOR RESPONDENTS WITH ONE OR MORE CHILDREN IN THE HOUSEHOLD (QUESTION 20)]

21. How old is the youngest child in your household?
(select age from drop-down menu)

Age	

[SHOWN TO RESPONDENTS WHO WORK (BASED ON QUESTION 2)]

22. How many hours do you work in an average week (incl. any overtime and homework)?
(select number of hours in drop-down menu)

Hours	

[SHOWN TO RESPONDENTS WHO WORK (BASED ON QUESTION 2)]

23. To what degree do you have the opportunity to plan when to work during the day?
(please tick only one box)

To a very high degree	❑
To a high degree	❑
To some degree	❑
To a lesser degree	❑
Not at all	❑

[SHOWN TO RESPONDENTS WHO WORK OR ARE UNDERGOING EDUCATION (BASED ON QUESTION 2)]

24. How far away from your job or place of study do you live?
(select the number of kilometres in the drop-down menu)

Kilometre	

25. The next questions concern whether your health makes it difficult to perform certain activities.
(please tick one box in each line)

	**No – not at all**	**Yes – somewhat difficult**	**Yes – very difficult**	**I cannot do that at all**
Do you have difficulty seeing (even if you wear glasses)?	❑	❑	❑	❑
Do you have difficulty hearing (even if you use a hearing aid)?	❑	❑	❑	❑
Do you have difficulty walking or walking the stairs?	❑	❑	❑	❑
Do you have difficulty remembering things or concentrating?	❑	❑	❑	❑
Do you have difficulty managing self-care such as washing yourself or getting dressed?	❑	❑	❑	❑
Do you have difficulty communicating in your preferred language, e.g. to understand others or to be understood?	❑	❑	❑	❑

26. Do you have any long-term health problems or disabilities?
(it is possible to tick multiple boxes)

Yes, a chronic disease (e.g. asthma, diabetes, sclerosis, cardiovascular disease, cancer, etc.)	❑
Yes, a mental disorder/behavioural disorder (e.g. anxiety, autism, ADHD, depression, concentration problems, etc.)	❑
Yes, a visual impairment (despite glasses or contact lenses)	❑
Yes, a hearing impairment (despite hearing aid or Cochlear Implant)	❑
Yes, a physical disability/handicap (e.g. amputation, cerebral palsy, rheumatoid disease, paralysis, etc.)	❑
Yes, a cognitive impairment (e.g. mental deficiency, speech impediment, etc.)	❑
Yes, another long-term health problem or disability (please specify)	❑
No	❑

[SHOWN ONLY TO RESPONDENTS WITH AT LEAST ONE LONG-TERM HEALTH PROBLEM OR DISABILITY (QUESTION 26). ONLY THE CATEGORY/CATEGORIES CHOSEN BY THE RESPONDENT ARE SHOWN]

In the previous question, you ticked one or more types of long-term health problems or disabilities. In this question, we ask you to indicate your specific health problems or disabilities.

27. What long-term health problems or disabilities do you have?
(it is possible to tick multiple boxes)

**Chronic disease**	
Allergy	❑
Asthma	❑
Bronchitis	❑
Epilepsy	❑
Rheumatoid disease	❑
HIV	❑
Skin disorder	❑
COPD	❑
Cancer	❑
Parkinson’s disease	❑
Problems with your heart, blood pressure or circulation	❑
Problems with your stomach, liver, kidneys or digestion	❑
Sclerosis	❑
Diabetes	❑
Difficulty in breathing	❑
Other chronic disease (please specify)	❑

**Mental disorder/behavioural disorder**	
ADHD, ADD	❑
Anxiety, phobia	❑
Autism, Asperger's disorder	❑
Depression, mania and bipolar disorder (manic-depressive)	❑
OCD	❑
Personality disorder, including Borderline	❑
PTSD (post-traumatic stress disorder)	❑
Eating disorder	❑
Hearing voices, schizophrenia and psychosis	❑
Stress, chronic fatigue syndrome, burnout	❑
Other mental/behavioural disorder (please specify)	❑

**Visual impairment**	
Blindness	❑
Weak-sighted (despite glasses or contact lenses)	❑
Severe weak-sightedness (despite glasses or contact lenses)	❑
Other visual impairment (please specify)	❑

**Hearing impairment**	
Deafness	❑
Reduced hearing (despite hearing aid or Cochlear Implant)	❑
Other hearing impairment (please specify)	❑

**Physical disability/handicap**	
Amputation	❑
Cerebral palsy	❑
Cystic fibrosis	❑
Epilepsy	❑
Acquired brain injury	❑
Fibromyalgia	❑
Rheumatoid disease	❑
Muscular dystrophy	❑
Osteoporosis (brittle-bone disease)	❑
Parkinson's disease	❑
Spina bifida	❑
Spinal cord injury	❑
Long-term side effects of polio	❑
Sclerosis	❑
Other physical disability (please specify)	❑

**Cognitive impairment**	
Acquired brain injury	❑
Speech impediment	❑
Mental deficiency (e.g. Down syndrome)	❑
Other cognitive impairment (please specify)	❑

[SHOWN ONLY TO RESPONDENTS WITH AT LEAST ONE LONG-TERM HEALTH PROBLEM OR DISABILITY (QUESTION 26)]

28. To what degree do your long-term health problems or disabilities hinder your ability to be physically active <u>in your leisure time</u>?
(please tick only one box)

To a very high degree	❑
To a high degree	❑
To some degree	❑
To a lesser degree	❑
Not at all	❑

29. How would you rate your current physical health in general?
(please tick only one box)

Very good	❑
Good	❑
Moderate	❑
Poor	❑
Very poor	❑

30. How would you rate your current mental health in general?
(please tick only one box)

Very good	❑
Good	❑
Moderate	❑
Poor	❑
Very poor	❑

31. Which of the following do you own or have daily access to?
(please tick one box in each line)

	**Yes**	**No**
Garden (incl. communal garden/courtyard)	❑	❑
Bike	❑	❑
Car	❑	❑
Dog	❑	❑

32. Has COVID-19/corona affected how often you have been physically active during the past 12 months? Indicate whether you have been more active, less active or if your level of activity is unchanged within each of the areas below.
(please tick one box in each line)

	Much more active	Slightly more active	Unchanged	Slightly less active	Much less active	Do not know/not relevant
Practical work at home	❑	❑	❑	❑	❑	❑
Physical activity at work or during your studies	❑	❑	❑	❑	❑	❑
Physical activity such as transport (e.g. walking, biking and running)	❑	❑	❑	❑	❑	❑
Physical activity during leisure time	❑	❑	❑	❑	❑	❑

[ITEM 'PHYSICAL ACTIVITY AT WORK OR DURING YOUR STUDIES' SHOWN TO RESPONDENTS WHO WORK (BASED ON QUESTION 2)'

33. If you have detailed information, suggestions and/or comments for the survey, please write them below.
[LARGE COMMENT FIELD]

[RECRUITMENT PAGE-1]
Thank you for completing the questionnaire. You have made a valuable contribution to 'Moving Denmark'.

We are going to conduct follow-up surveys in the near future, and we hope that we may contact you again. If you are interested in participating in one or more of the surveys, please indicate so below. You will have the opportunity to say no at a later time.

I would like to be contacted regarding possible participation in:
(please tick one box in each line)

	Yes	No
An interview about my exercise habits	❑	❑
A measurement of my daily exercise for a week with an activity meter	❑	❑
A map-based questionnaire about the places I am physically active	❑	❑
A panel that will receive a questionnaire about exercise habits every 4-5 years	❑	❑

[SHOWN ONLY TO RESPONDENTS WHO HAVE ANSWERED 'YES' TO BE CONTACTED FOR AT LEAST ONE MEASUREMENT]
[RECRUITMENT PAGE-2]

If you are selected to participate in the follow-up surveys, we will contact you by email. For this we need your email address.
(write your email below)

____

If you do not have an email address, please provide a telephone number instead where we can reach you.
(write your phone number below)

____

[END PAGE]
Thanks again for participating in the survey.

If you would like to print your answers, please click the printer icon below.

When you click 'Afslut' (Finish), you will be taken to the website for 'Moving Denmark'. Here you can read more about the project. Results from the survey will also be published there.
