# Supplementary material for: The association between the frequency of physical activity and social background within and across home, occupational, transportation and leisure domains
Source: Front Sports Act Living. 2025 Aug 12;7:1609485. doi: 10.3389/fspor.2025.1609485 (PMC12378642; doi:10.3389/fspor.2025.1609485)
Supplement: Supplementary file 2 [file Datasheet2.pdf]

Supplementary file 2: Overview of the activities from the questionnaire, categorization of the activity groups within the domains.

Activities (26)	Activity groups (11)	Domains (4)
Cooking, tidying up and dishwashing	Home indoor	Home
Cleaning		
Laundry		
Gardening	Home outdoor	
Other practical work		
Work/study tasks with lighter physical exertion	Occupational PA[a]	Occupational
Work/study tasks with moderate physical exertion		
Work/study tasks with hard physical exertion		
Biking to/from work/study	Biking as transportation	Transportation
Biking to/from other tasks		
Walking to/from work/study	Walking as transportation	
Walking to/from other tasks		
Walking- and hiking<br>(not as transportation –in high/moderate pace, e.g. walking with dog)	Walking/biking	Leisure time
Biking<br>(not as transportation – e.g. road cycling, mountain biking)		
Running<br>(not as transportation – trail running, cross-country)	Running/fitness/flexibility training	
Fitness and physical training<br>(e.g. strength exercises/training, cardiovascular training, aerobics, CrossFit, spinning other group training)		
Mental/flexibility/stability training<br>(e.g. yoga, Pilates, meditation/mindfulness, tai chi, qi gong)		
Team ballgames<br>(e.g. basketball, football/soccer, hockey/floorball, handball, volleyball/beach volleyball)	Ballgames	
Other ballgames<br>(e.g. badminton, table tennis, golf, paddle tennis, squash, tennis)		
Gymnastics<br>(e.g. artistic/rhythmic gymnastics, apparatus gymnastics, fitness gymnastics, trampoline)	Gymnastics/dance	
Dance<br>(e.g. partner dancing, ballet/flamenco/modern, fitness dance, creative dance, street dance)		
Activities in water<br>(e.g. swimming in indoor pool, open water swimming, diving/free diving/underwater hunting, pool training, winter swimming)	Water/outdoor activities	
Activities on water<br>(e.g. canoeing/kayaking, rowing, sailing, windsurfing/kitesurfing, surfing/stand up paddle boarding)		
Outdoor activities<br>(e.g. outdoor life, hunting, fishing, scouting, live action role-playing outdoor)		
Street sports (e.g. calisthenics/street workout, scooter/BMX, parkour/free running/tricking, roller skating, skate-/long-/carver boarding, street basketball/soccer/panna)	Other sport/street activities	
Other sport activities (e.g. athletics, billiards/pool, bowling, archery, martial arts, petanque, equestrianism, roller skiing, skiing, shooting, triathlon/duathlon)		

[a] *PA = Physical Activity.*
